# Birth weight, gestational age, fetal growth and childhood asthma hospitalization

**DOI:** 10.1186/1710-1492-10-13

**Published:** 2014-03-06

**Authors:** Xiaoqin Liu, Jørn Olsen, Esben Agerbo, Wei Yuan, Sven Cnattingius, Mika Gissler, Jiong Li

**Affiliations:** 1 Section for Epidemiology, Department of Public Health, Aarhus University, Aarhus, Denmark; 2 Department of Epidemiology and Social Science on Reproductive Health, Shanghai Institute of Planned Parenthood Research, WHO Collaborating Center for Research in Human Reproduction, National Population & Family Planning Key Laboratory of Contraceptive Drugs and Devices, Shanghai, China; 3 Department of Epidemiology, Fielding School of Public Health, University of California, Los Angeles, CA, USA; 4 National Centre for Register-Based Research, Aarhus University, Aarhus, Denmark; 5 CIRRAU-Centre for Integrated Register-based Research, Aarhus University, Aarhus, Denmark; 6 Clinical Epidemiology Unit, Department of Medicin Solna, Karolinska University Hospital, Karolinska Institute, Stockholm, Sweden; 7 Information Department, THL National Institute for Health and Welfare, Helsinki, Finland; 8 NHV Nordic School of Public Health, Gothenburg, Sweden

**Keywords:** Asthma, Birth weight, Gestational age, Hospitalization, Small for gestational age

## Abstract

**Background:**

Childhood asthma may have a fetal origin through fetal growth and development of the immunocompetence or respiratory organs.

**Objective:**

We examined to which extent short gestational age, low birth weight and fetal growth restriction were associated with an increased risk of asthma hospitalization in childhood.

**Methods:**

We undertook a cohort study based on several national registers in Denmark, Sweden and Finland. We included all live singleton born children in Denmark during 1979-2005 (N = 1,538,093), in Sweden during 1973-2004 (N = 3,067,670), and a 90% random sample of singleton children born in Finland during 1987-2004 (N = 1,050,744). The children were followed from three years of age to first hospitalization for asthma, emigration, death, their 18th birthday, or the end of study (the end of 2008 in Denmark, and the end of 2007 in Sweden or Finland), whichever came first. We computed the pseudo-values for each observation and used them in a generalized estimating equation to estimate relative risks (RR) for asthma hospitalization.

**Results:**

A total of 131,783 children were hospitalized for asthma during follow-up. The risk for asthma hospitalization consistently increased with lower birth weight and shorter gestational age. A 1000-g decrease in birth weight corresponded to a RR of 1.17 (95% confidence interval (CI) 1.15-1.18). A one-week decrease in gestational age corresponded to a RR of 1.05 (95% CI 1.04-1.06). Small for gestational age was associated with an increased risk of asthma hospitalization in term but not in preterm born children.

**Conclusions:**

Fetal growth and gestational age may play a direct or indirect causal role in the development of childhood asthma.

## Introduction

Asthma is a significant health problem for families and society [1]. More than 50% of children with asthma develop symptoms before their fifth birthday [2], indicating that pre- or perinatal factors may play a causal role.

Preterm birth, low birth weight and fetal growth restriction may be associated with disturbed immunocompetence and restrict normal lung growth and development [3-6], thereby predispose children to asthma later in life [4,7]. However, epidemiologic findings linking gestational age, birth weight, fetal growth and childhood asthma are inconsistent. Gestational age has been associated with asthma in some studies [8-17] but not in others [18-23]. Previous studies on birth weight and asthma are also contradictory with both inverse [8,9,13,14,21,23-26], direct [18,27], as well as null results [19,20,28,29]. As gestational age and birth weight are strongly correlated, small for gestational age (SGA) is commonly used as a proxy for fetal growth restriction [30]. Findings on asthma and SGA have also been inconclusive. While some indicated an association between SGA and increased risk of asthma [15,31], others found no association [9,32,33], or even an inverse association [34,35]. Comparison of these studies is hampered by differences in defining the study populations and their size, e.g. children born with different gestational age and different follow-up time periods. Results from twin studies may not reflect associations for singletons [19,23-25]. Studies that found no association were often based on smaller sample sizes [18,20,33]. And there has currently been no “gold standard” for diagnosis of asthma and it remains unclear how the condition should be defined and measured in epidemiological studies. Self-reported asthma may be subject to recall bias and subjective interpretation of disease status [19-22,25,29,33]. Asthma diagnosis is considered less accurate in young children due to its clinical instability in early years of life [33,36,37]. In addition, different reference curves for birth weight for gestational age have been used to estimate fetal growth [38]. Further, these curves are usually based on the distribution of live births and do not necessarily reflect normal fetal growth. Preterm born infants are smaller than fetuses of the same gestational age due to the fact that fetal growth restriction is a risk factor for both medically indicated and spontaneous preterm birth [39].

Large population-based studies using the register systems in three Nordic countries allowed a detailed analysis of the association between gestational age, birth weight, fetal growth and risk of childhood asthma. We included information on a number of social demographic variables and explored the risk for asthma hospitalization as a function of gestational age, birth weight, and fetal growth in a cohort of children followed until they reached 18 years of age.

## Methods

### Study population

We used a cohort study based on linkage between several national registers in Denmark, Sweden and Finland. All live-born children and new residents in the three Nordic countries are assigned a unique personal identification number, which can be used to link information at the individual level in all national registers. We first identified all singleton live births during 1979-2005 recorded in the Danish Medical Birth Registry (DMBR) and during 1973-2004 in the Swedish Medical Birth Register (SMBR). In Finland, we were only allowed to include a 90% random sample of singleton children born during 1987-2004 in the Finnish Medical Birth Registry (FMBR). The DMBR has been computerized since 1973 and includes data on gestational age from 1978. The SMBR was established in 1973, and the FMBR was established in 1987. The DMBR was linked to the Danish Civil Registration System, the Danish National Patient Register [40], and the Danish Integrated Database for Longitudinal Labor Market Research. The SMBR was linked to the Swedish Multi-generation Register, the Swedish Patient Register [41], and the Swedish Education Register. The FMBR was linked to the Finnish Hospital Discharge Register [42] and the Population Register at Statistics Finland. A total of 5,928,759 births were recorded during the study period in the birth registries. We excluded 198,733 (3.3%) infants who had missing or unrealistic gestational age or birth weight data (gestational age < 154 or > 315 days and birth weight < 300 or > 6400 grams), 44 infants whose mothers’ data on parity were missing, 30,424 deceased infants and 43,051 infants who emigrated under three years of age. Seven infants were reported to die from asthma before three years of age. Of remaining 5,656,507 children, 1,538,093 were born in Denmark (27.2%), 3,067,670 in Sweden (54.2%), and 1,050,744 in Finland (18.6%).

### Exposures

We used three different indicators of fetal development and growth: gestational age, birth weight, and SGA. Information on birth weight and gestational age were obtained from the DMBR, SMBR and FMBR. Gestational age in the DMBR was previously based on the date of last menstrual period (LMP), but in the recent 20 years, ultrasound measurements have been increasingly used to correct LMP if needed. In Sweden, early second trimester ultrasonography to estimate gestational age is routinely offered since 1990, and 95% of women accept this offer, otherwise the date of the LMP is used. Gestational age in the FMBR was estimated from the date of the LMP, unless there was a discrepancy with the first-or second-trimester ultrasonography measurements of more than seven or 14 days, respectively, in which case the latter measurements were used. We categorized gestational age at birth into six groups: 22-28 weeks, 29-32 weeks, 33-36 weeks, 37-38 weeks, 39-41 weeks, and 42-45 weeks. We categorized birth weight into 10 groups: <1000 g, 1000-1499 g, 1500-1999 g, 2000-2499 g, 2500-2999 g, 3000-3499 g, 3500-3999 g, 4000-4499 g, 4500-4999 g, and ≥5000 g. Expected birth weight was calculated using the sex-specific fetal growth curves for gestational age by Marsal et al. [43]. SGA was defined as a birth weight < -2 SD of expected birth weight, large for gestational age (LGA) as >2 SD of expected birth weight [43], and appropriate for gestational age (AGA) as ≥ -2 SD and ≤2 SD of expected birth weight. Z-scores were calculated using the following formula: Z-score = (birth weight-expected birth weight according to gestational age and sex)/SD of expected birth weight according to gestational age and sex.

### Outcome

Diagnosis of asthma in young children aged 0-3 years is difficult because asthma symptoms are often non-specific [44]. Self-reported asthma cannot be validated against a medical diagnosis because of recall bias and individual differences in symptom perception [45]. Using population-based register data to obtain information about asthma can be useful as they do not depend on recall but upon routinely medically diagnosed asthma cases. For that reason, we used hospitalization for asthma after three years of age, as recorded in the Danish National Hospital Register, the Swedish Patient Register, and the Finnish Hospital Discharge Register. The Danish National Patient Registry includes inpatient diagnosis on all hospitalizations in the country since 1977; outpatient diagnoses were included from 1995 onwards [40]. The Swedish Patient Register has collected information on inpatient care since 1964/1965 and reached nation-wide coverage in 1987 [41]. The Finnish Hospital Discharge Register contains nationwide linkable data on all inpatient hospital discharges since 1969 and all outpatient visits to public hospitals since 1998 [42]. Asthma was identified based on the following International Classification of Diseases (ICD) codes: 493 (ICD-8 and ICD-9); and J45, J46 (ICD-10). The first hospitalization for asthma was defined as the date of first admission in the registers for those who had one of the above mentioned ICD codes.

### Follow-up

The children were followed from three years of age to hospitalization for asthma, emigration, death, their 18th birthday, or the end of follow up (the end of 2008 in Denmark, and the end of 2007 in Sweden or Finland), whichever came first.

### Statistical analysis

We measured the cumulative risk for asthma hospitalization, using STATA (version 11.2). A method based on pseudo-values has been proposed for direct regression modeling of cumulative incidence function with right censored data at a fixed point in time [46], which was 18 years of age in our study. The pseudo-values were calculated for each individual and generated once. We computed the pseudo-values for each observation based on the difference between the complete sample and the leave-one-out estimators of relevant survival quantities. All computations were performed in STATA using the stpsurv command for generating the pseudo-observations for the failure function [46]. We used these pseudo-values in a generalized estimating equation (GEE) to model the effects of fetal development on asthma hospitalization with a log link function. The relative risk (RR) for asthma hospitalization was estimated with 95% confidence interval (CI). All multivariable models were adjusted for potential confounders, including country of residence (Denmark, Sweden, and Finland), maternal age at delivery (15-26 years, 27-30 years, ≥ 31 years), parity (1st, 2nd, 3rd and higher), mode of delivery (delivered vaginally, delivered by cesarean section), maternal social status at birth (not in labor market, unskilled workers, skilled workers and white collar workers, top level status), family history of asthma (in father, mother or sibling), sex of the children, and calendar year of birth (1973-1977, 1978-1982, 1983-1987, 1988-1992, 1993-1997, 1998-2002, 2003-2005).

We explored the association between gestational age, birth weight, SGA and asthma hospitalization separately. When we focused on predictors as recorded at birth, we did the analysis by including gestational age and birth weight in the same model. In order to explore whether the association between fetal growth and asthma hospitalization changed with gestational age, we also performed the analysis stratified by gestational age. The association between birth weight and asthma risk was explored using 3500-3999 g as a reference. We used full term (39-41 weeks) children as the reference group when analyzing the association between gestational age and hospitalization for asthma. The association between estimated fetal growth and asthma risk was explored using AGA children as reference.

As cesarean section has been proposed to affect immune system development [47], we did a sub-analysis stratified on mode of delivery. In order to find whether fetal development acts on asthma hospitalization through complications of preterm birth, we did a sub-analysis using Danish and Finnish registers by further adjustment for complications of preterm birth (bronchopulmonary dysplasia and respiratory distress syndrome). We also did a subgroup analysis to estimate residual confounding by linking to the Danish National Birth Cohort [48]. In addition to aforementioned potential confounders, the confounding effect of weight before pregnancy, maternal asthma during pregnancy, maternal smoking during pregnancy, breastfeeding duration, environmental tobacco smoke (ETS) exposure, contact with pets in childhood, mother’s marital status, and cohabit status was estimated in this subsample.

To examine whether associations between fetal development and onset of asthma were dependent on the asthma definition, we also replicated our analyses by identifying asthma on the basis of asthma medications in live singleton births during 1993-2005 recorded in the DMBR. Information on asthma medication was obtained from the Danish National Prescription Registry [49]. The anatomical therapeutical chemical (ATC) codes for inhaled asthma drugs were: inhaled β_2_-agonists (R03AC02, R03AC03, R03AC04, R03AC12 and R03AC13), inhaled glucocorticoids (R03BA01, R03BA02 and R03BA05), fixed-dose combination of inhaled β_2_-agonists and glucocorticoids (R03AK06 and R03AK07), and leukotriene receptor antagonists (R03DC03). We defined asthma according to at least two prescriptions of asthma drugs after three years of age. Medications prescribed on the same day were considered to represent one prescription. The first asthma occurrence was defined as the date of first anti-asthmatic drugs redeemed.

### Ethics

The study was approved by Danish Data Protection Agency and Scientific Ethics Committee of Central Region Jutland in Denmark and Research Ethics Committee (EPN) at Karolinska Institute in Sweden, Statistics Finland and National Institute for Health and Welfare (THL) in Finland. No informed consent is needed for register-based study based on encrypted data according to the legislation in Denmark, Sweden, and Finland.

## Results

The mean follow-up time was 10.6 years (95% CI 1.1-15.0), altogether 6.0 × 10^7^ person-years. A total of 131,783 children were hospitalized at least once for asthma during the study period. Table 1 shows the characteristics of the study population.

**Table 1:** Characteristics of study population according to estimated fetal growth

**Characteristics**	**N**	**SGA**	**AGA**	**LGA**
Gestational age				
22-28 weeks	10,632	21.5	72.5	6.0
29-32 weeks	31,964	22.0	72.4	5.6
33-36 weeks	218,432	8.5	85.4	6.1
37-38 weeks	934,450	3.1	91.3	5.6
39-41 weeks	4,010,066	2.3	94.7	3.0
42-45 weeks	450,963	4.4	94.2	1.4
Birth weight				
<1000 g	7,633	54.3	44.9	0.8
1000-1499 g	17,555	49.3	50.1	0.6
1500-1999 g	36,768	45.9	53.7	0.4
2000-2499 g	123,725	42.2	57.5	0.3
2500-2999 g	598,400	13.5	86.3	0.2
3000-3499 g	1,828,077	0.3	99.5	0.2
3500-3999 g	1,995,356	0	99.2	0.8
4000-4499 g	855,601	0	91.8	8.2
4500-4999 g	170,630	0	50.7	49.3
≥5000 g	22,762	0	4.1	95.9
Country of residence				
Denmark	1,538,093	3.5	93.0	3.5
Sweden	3,067,670	3.0	93.8	3.2
Finland	1,050,744	2.2	93.6	4.2
Maternal age at delivery				
15-26 years	2,089,571	3.4	94.1	2.5
27-30 years	1,604,838	2.7	93.9	3.4
≥31 years	1,961,791	2.7	92.7	4.6
Unknown	307	5.9	91.2	2.9
Maternal social status				
Not in labor market	836,704	3.3	93.3	3.4
Unskilled workers	1,131,613	3.0	93.5	3.5
Skilled workers and white collar workers	1,591,435	2.8	93.7	3.5
Top level status	778,580	2.8	93.6	3.6
Unknown	1,318,175	3.0	93.5	3.5
Parity				
1	2,332,141	4.1	93.9	2.0
2	2,018,981	2.2	93.9	3.9
≥3	1,305,385	2.2	92.3	5.5
Mode of delivery				
Delivered vaginally	5,020,529	2.5	94.3	3.2
Delivered by cesarean section	635,077	6.7	87.4	5.9
Unknown	901	3.6	92.2	4.2
Sex of the child				
Boy	2,902,138	2.9	93.5	3.6
Girl	2,754,369	3.0	93.6	3.4
Calendar year of birth				
1973-1977	493,193	4.5	93.0	2.5
1978-1982	629,589	3.8	93.6	2.6
1983-1987	777,959	3.3	93.7	3.0
1988-1992	1,178,159	2.8	93.6	3.6
1993-1997	1,106,229	2.5	93.5	4.0
1998-2002	995,480	2.5	93.6	3.9
2003-2005	475,898	2.4	93.7	3.9
Family history of asthma				
Yes	416,278	3.7	92.8	3.5
No	5,240,229	2.9	93.6	3.5

**Figure 1:**
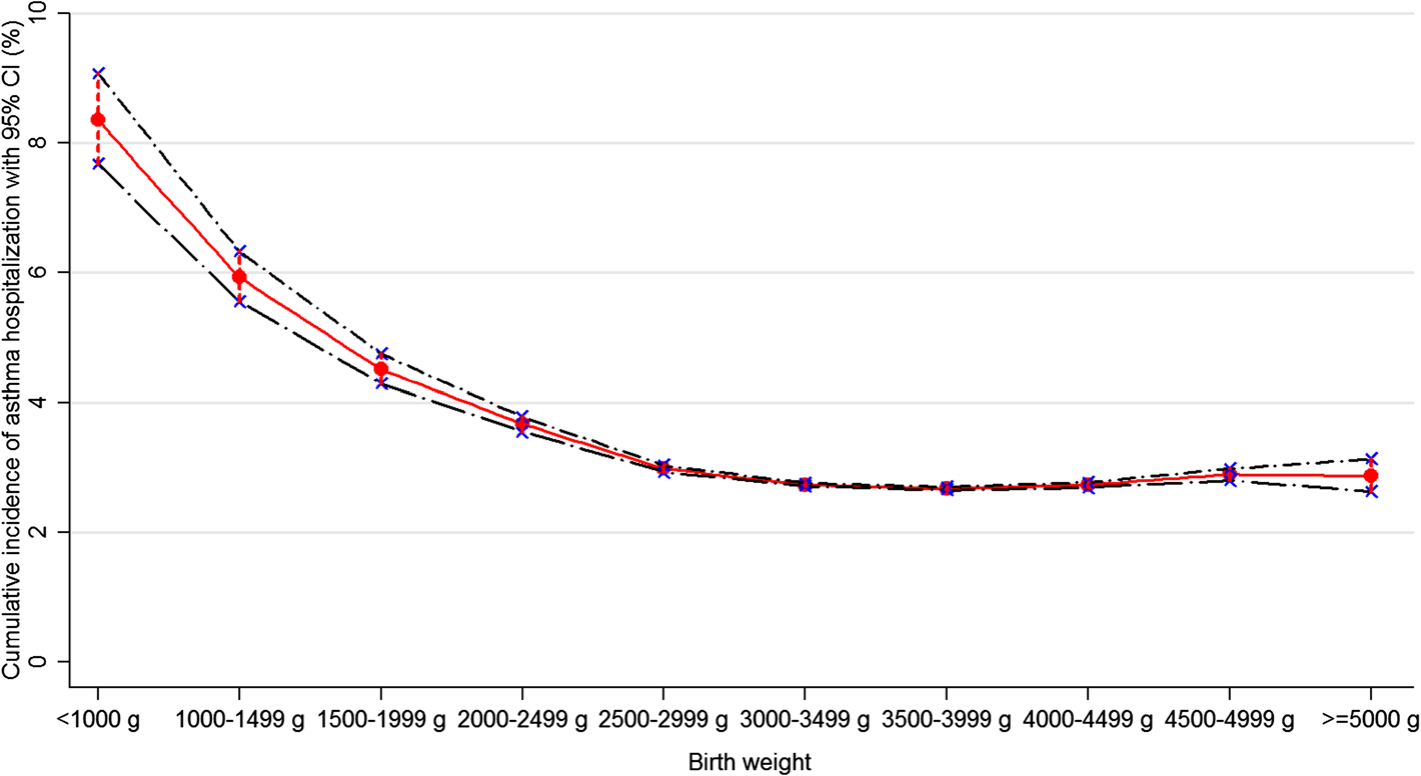
Cumulative incidence of asthma hospitalization with 95% CI according to birth weight.

**Figure 2:**
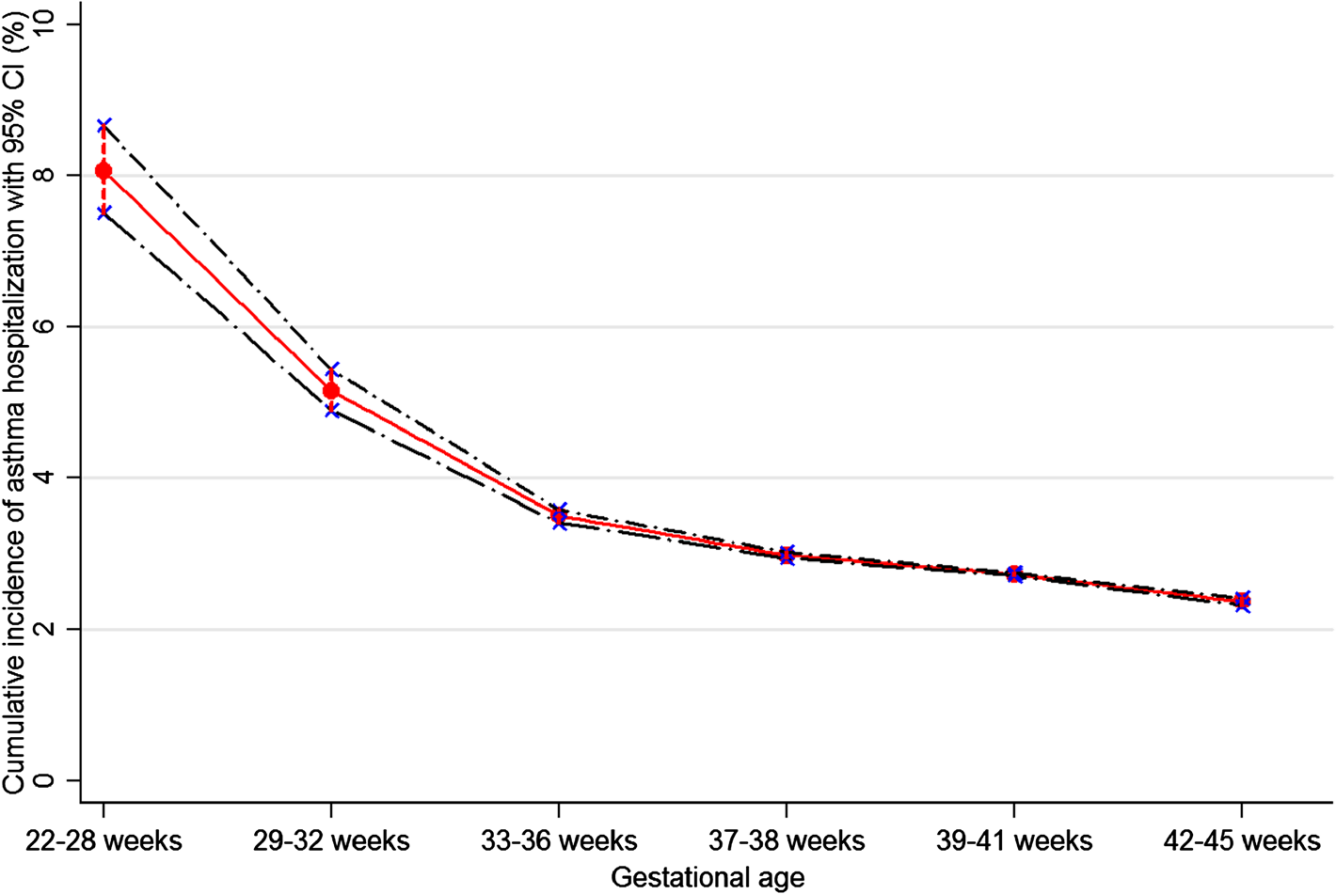
Cumulative incidence of asthma hospitalization with 95% CI according to gestational age.

The risk of hospitalization for asthma increased with lower birth weight and shorter gestational age. The highest cumulative incidence was found in children born with a birth weight of < 1000 g and extremely preterm births (children born at 22-28 weeks) (Figures 1 and 2). A 1000-g decrease in birth weight corresponded to a RR of 1.17 (95% CI 1.15-1.18). Compared with full term (39-41 weeks) children, the adjusted RR of asthma hospitalization born at 22-28 weeks was 2.26 (95% CI 2.10-2.43). Even early term (37-38 weeks) children had a higher risk of hospitalization for asthma than full term children (RR 1.10, 95% CI 1.09-1.12). A one-week decrease in gestational age corresponded to a RR of 1.05 (95% CI 1.04-1.06). Children born SGA were associated with a moderately increased risk of asthma hospitalization (RR 1.20, 95% CI 1.16-1.24). Risk for asthma increased steadily with decreasing Z-score for children (RR 1.03, 95% CI 1.02-1.04) (Table 2). When analyzing predictors for asthma hospitalization, we included gestational age and birth weight in the same model. The RR was 1.08 (95% CI 1.06-1.09) for a 1000-g decrease in birth weight and 1.04 (95% CI 1.03 -1.05) for a one-week decrease in gestational age.

**Table 2:** RR for asthma hospitalization in childhood according to birth weight, gestational age, and fetal growth

**Fetal development variables**	**Cases**	**Cumulative incidence (%)**	**Crude RR**	Adjusted RR* (95% CI)
Birth weight				
<1000 g	542	8.35	3.03	2.33 (2.14-2.53)
1000-1499 g	897	5.93	2.21	1.81 (1.68-1.95)
1500-1999 g	1,423	4.51	1.69	1.48 (1.40-1.57)
2000-2499 g	3,823	3.67	1.38	1.33 (1.28-1.38)
2500-2999 g	14,992	2.98	1.12	1.15 (1.12-1.17)
3000-3499 g	41,716	2.73	1.02	1.05 (1.04-1.07)
3500-3999 g	44,451	2.67	1	1 (ref)
4000-4499 g	19,371	2.73	1.02	0.99 (0.97-1.01)
4500-4999 g	4,037	2.89	1.08	1.03 (0.99-1.06)
≥5000 g	531	2.87	1.08	1.01 (0.92-1.11)
Birth weight per 1000-g decrease	-	-	1.18	1.17 (1.15-1.18)
Gestational age				
22-28 weeks	737	8.07	2.89	2.26 (2.10-2.43)
29-32 weeks	1,416	5.16	1.88	1.63 (1.54-1.73)
33-36 weeks	6,471	3.50	1.28	1.26 (1.22-1.29)
37-38 weeks	23,062	2.98	1.09	1.10 (1.09-1.12)
39-41 weeks	91,055	2.73	1	1 (ref)
42-45 weeks	9,042	2.37	0.86	0.98 (0.95-1.00)
GA per reduced week	-	-	1.07	1.05 (1.04-1.06)
Fetal growth				
SGA	4,878	3.38	1.23	1.20 (1.16-1.24)
AGA	121,741	2.76	1	1 (ref)
LGA	5,164	3.18	1.15	1.05 (1.02-1.09)
Z-score per decreased of SD	-	-	1.00	1.03 (1.02-1.04)

*Adjusted for country of residence, maternal age at delivery, maternal social status, parity, mode of delivery, sex of the child, calendar year of birth and family history of asthma.

We estimated the association between fetal growth and asthma hospitalization stratified by gestational age. There was a significant association between SGA and increased risk of asthma in term but not in preterm born (<37 weeks) children. However, with the exception of extremely preterm born children (22–28 weeks) and children born after 42 gestational weeks, the risk of asthma increased with decreasing Z-sore in all preterm and term gestational age groups. In extremely and moderately preterm born children (22-32 weeks), LGA children had a decreased risk of asthma hospitalization (Table 3).

**Table 3:** RR for asthma hospitalization in childhood according to gestational age and estimated fetal growth

**Fetal development variables**	**Cases**	**Cumulative incidence (%)**	**Crude RR**	Adjusted RR* (95% CI)
22-28 weeks				
SGA	180	9.09	1.14	1.02 (0.86-1.21)
AGA	527	7.96	1	1 (ref)
LGA	30	5.43	0.71	0.71 (0.46-1.11)
Z-score per decreased of SD	-	-	1.04	1.02 (0.99-1.04)
29-32 weeks				
SGA	359	6.15	1.20	1.07 (0.94-1.22)
AGA	1,010	5.05	1	1 (ref)
LGA	47	2.96	0.58	0.60 (0.43-0.85)
Z-score per decreased of SD	-	-	1.06	1.04 (1.02-1.07)
33-36 weeks				
SGA	675	4.27	1.26	1.06 (0.97-1.17)
AGA	5,409	3.43	1	1 (ref)
LGA	387	3.41	0.99	0.94 (0.84-1.05)
Z-score per decreased of SD	-	-	1.05	1.03 (1.01-1.04)
37-38 weeks				
SGA	860	3.56	1.22	1.15 (1.07-1.24)
AGA	20,735	2.93	1	1 (ref)
LGA	1,467	3.43	1.17	1.03 (0.97-1.09)
Z-score per decreased of SD	-	-	0.99	1.02 (1.01-1.03)
39-41 weeks				
SGA	2,349	3.01	1.12	1.12 (1.07-1.17)
AGA	85,611	2.71	1	1 (ref)
LGA	3,095	3.06	1.13	1.06 (1.02-1.10)
Z-score per decreased of SD	-	-	0.99	1.02 (1.01-1.03)
42-45 weeks				
SGA	455	2.51	1.07	1.11 (0.98-1.25)
AGA	8,449	2.36	1	1 (ref)
LGA	138	2.83	1.20	1.15 (0.95-1.40)
Z-score per decreased of SD	-	-	1.00	1.02 (0.99-1.05)

*Adjusted for country of residence, maternal age at delivery, maternal social status, parity, mode of delivery, sex of the child, calendar year of birth and family history of asthma.

The associations were similar in children who were delivered vaginally and by cesarean section (data not shown). The associations between low birth weight, short gestational age, SGA, and risk of asthma hospitalization attenuated slightly by further adjustment for complications of preterm birth but remained statistically significant (data not shown). In sub-analysis, after further adjustment for weight before pregnancy, maternal asthma during pregnancy, maternal smoking during pregnancy, breastfeeding duration, ETS exposure, contact with pets, mother’s marital status, and cohabit status, the estimated RRs were similar to those obtained without adjustment for these potential confounders (data not shown).

When the outcome was asthma medication, the aforementioned associations remained statistically significant although the magnitude of associations decreased slightly (Table 4).

**Table 4:** RR for asthma hospitalization and medication in childhood according to birth weight, gestational age and fetal growth in Denmark, 1993-2005

**Fetal development variables**	**Asthma hospitalization**				**Asthma medication**			
	**Cases**	**Cumulative incidence (%)**	**Crude RR**	Adjusted RR* (95% CI)	**Cases**	**Cumulative incidence (%)**	**Crude RR**	Adjusted RR* (95% CI)
Birth weight								
<1000 g	94	13.58	2.64	2.82 (2.12-3.74)	306	34.06	2.00	1.94 (1.75-2.15)
1000-1499 g	184	9.01	1.91	1.71 (1.42-2.06)	624	27.12	1.63	1.51 (1.39-1.65)
1500-1999 g	318	7.76	1.64	1.41 (1.21-1.64)	1,107	24.23	1.45	1.37 (1.28-1.47)
2000-2499 g	812	6.87	1.46	1.30 (1.17-1.44)	2,950	22.30	1.34	1.29 (1.22-1.36)
2500-2999 g	2,919	5.29	1.12	1.10 (1.03-1.18)	11,142	18.79	1.13	1.11 (1.07-1.16)
3000-3499 g	8,099	5.01	1.06	1.07 (1.02-1.13)	30,145	16.89	1.02	1.01 (0.98-1.04)
3500-3999 g	8,500	4.72	1	1 (ref)	32,242	16.43	1	1 (ref)
4000-4499 g	4,045	4.89	1.04	1.01 (0.95-1.08)	14,984	16.55	1.00	0.99 (0.95-1.02)
4500-4999 g	869	4.74	1.01	1.02 (0.92-1.12)	3,165	16.59	1.00	1.00 (0.95-1.05)
≥5000 g	135	5.56	1.18	1.15 (0.91-1.45)	463	16.48	1.01	0.99 (0.89-1.08)
Birth weight per 1000-g decrease	-	-	1.17	1.15 (1.11-1.19)	-	-	1.13	1.11 (1.10-1.13)
Gestational age								
22-28 weeks	129	11.72	2.34	2.37 (1.87-2.99)	412	30.69	1.86	1.78 (1.64-1.94)
29-32 weeks	301	8.65	1.79	1.49 (1.29-1.72)	1,084	25.67	1.56	1.45 (1.36-1.54)
33-36 weeks	1,318	6.36	1.32	1.20 (1.10-1.30)	5,021	21.90	1.33	1.26 (1.22-1.30)
37-38 weeks	4,464	5.67	1.17	1.14 (1.08-1.21)	16,967	18.69	1.13	1.10 (1.08-1.13)
39-41 weeks	17,616	4.79	1	1 (ref)	65,682	16.44	1	1 (ref)
42-45 weeks	2,147	4.61	0.96	0.94 (0.88-1.01)	7,962	17.40	1.05	1.05 (0.95-1.16)
GA per reduced week	-	-	1.06	1.05 (1.04-1.06)	-	-	1.04	1.04 (1.03-1.05)
Birth weight for gestational age								
SGA	1,055	6.36	1.29	1.20 (1.10-1.31)	3,776	21.15	1.25	1.19 (1.14-1.24)
AGA	23,784	4.94	1	1 (ref)	89,263	17.02	1	1 (ref)
LGA	1,136	5.32	1.07	1.11 (0.99-1.24)	4,089	17.34	1.02	1.04 (0.99-1.09)
Z-score per decreased of SD	-	-	1.03	1.01 (0.99-1.03)	-	-	1.03	1.02 (1.01-1.03)

*Adjusted for maternal age at delivery, maternal social status, parity, mode of delivery, sex of the child, calendar year of birth and family history of asthma.

## Discussion

Our results indicated that risk for asthma hospitalization increased with lower birth weight and shorter gestational age. Even early term (37-38 weeks) children had a higher risk of hospitalization for asthma than full term (39-41 weeks) children. SGA was associated with a slightly increased risk of hospitalization for asthma in term but not in preterm born children. LGA was associated with a decreased risk of asthma hospitalization in extremely and moderately preterm born children.

The inverse association we found between birth weight and asthma hospitalization concurs with most previous cohort studies [8,9,14,21,23-26]. Low birth weight often results from fetal growth restriction, preterm birth or both. Low birth weight may act as a mediating factor between the determinants of low birth weight and asthma hospitalization. Thus, these associations may reflect unknown causes of asthma also influencing fetal growth and/or length of gestation.

In the present study, an increased risk of hospitalization for asthma was associated with decreasing gestational age. The finding is consistent with most [8-17], although not all [18-22], previous cohort studies, which may reflect instable results due to small sample sizes [18-22]. Wheezing symptoms in children under three years are common and often transient [50], and including children with transient wheezes in the asthma group may dilute possible associations specific to asthma [51]. The inconsistent finding may reflect that the association we found is mainly seen for severe cases. The preterm birth may be related to a deficit in the structure and function of the lung at birth [3], which may increase the risk of subsequent asthma development. It is also possible that preterm birth and asthma share common genetic determinants or environmental exposures. For instance, studies have indicated that maternal asthma is associated with both preterm birth and childhood asthma [9,52].

We also found that early term children had a higher risk of hospitalization for asthma compared with full term children [17]. Although the relative risk of hospitalization for asthma for early term children is lower than for preterm children, the larger number of children born at early term may present a greater disease burden in the population.

Evidence linking fetal growth restriction to childhood asthma is limited. In the present study, SGA was associated with a slightly increased risk of hospitalization for asthma. Our findings are in concordance with two of three cohort studies [15,24], but not with an early study [9]. Several studies have limited power to detect the modest association we observed [32,33,53,54]. And cross-sectional studies may be vulnerable to recall bias [14,16,17]. As preterm born infants are smaller than fetuses of the same gestational age [39], studies, categorizing SGA based on the distributions of live births [32,33,53] may misclassify SGA as AGA, and thereby dilute a possible association. Two previous studies explored the association between fetal growth and asthma using measured fetal growth as a predictor for asthma [9,54], with null findings. One study had limited power to detect the modest association we observed due to smaller sample size [54]. Use of a broader asthma definition may lead to more non-differential misclassification and therefore dilute the association [9]. The conflicting findings may also suggest that the association between fetal growth and hospitalization for asthma is weak, or is caused by factors that correlate with fetal growth. Normal lung development depends on the presence of appropriate oxygen tension and nutrition. Children born with fetal growth restriction have a greater risk of developing brochopulmonary dysplasia [4], which is associated with childhood asthma [55], providing a potential mechanism by which fetal growth restriction may increase the risk of asthma. However, this probably does not fully explain the association we observed as the association attenuated after further adjustment for the respiratory complications of preterm birth but remained statistically significant. Factors responsible for fetal growth restriction may also lead to “programming” of the respiratory or immune systems [56], predisposing children to the development of asthma. The association between SGA and hospitalization for asthma was observed in term but not in preterm born children. The underlying mechanism is not clear. Further studies elucidating the mechanisms are warranted.

A novel finding in our study was LGA was associated with a deceased risk for asthma hospitalization in extremely and moderately preterm born children. Our result is consistent with one previous study showing the association of high birth weight for gestational age and a decreased risk of asthma in preterm children [24]. Our data support the previous study that has found a reduced risk of chronic lung disease in children born large for gestational age and preterm, compared with children born appropriate for gestational age and preterm [4].

Our study is the largest study so far on this topic. The large sample size allowed us to analyze the association between birth characteristics and child hospitalization for asthma with almost complete follow-up.

Our study also has limitations. We did not investigate confounding from familial lifestyle and environmental factors, including housing status, early exposure to allergens, diet and nutrition. We used first hospital discharge diagnosis after three years of age as the outcome, and therefore, our findings addressed severe asthma or factors that may lower the threshold for asthma hospitalization. Although we did a sub-analysis with asthma medication and got similar findings, we did not include all patients with mild symptoms who did not seek medical help. Therefore, our findings cannot be generalized to those children as well as children with transient asthma. Second, children born with low gestational age or fetal growth restriction may have more opportunities to have asthma diagnosed because they are hospitalized more frequently due to co-morbidity [57]. Our findings were similar to those reported in other large cohort studies using different definitions of asthma [15,24]. In a sub-analysis, we compared findings using two different criteria to identify cases: prescription data and hospitalization data. We found that short gestational age and fetal growth restriction increased the risk of asthma regardless of data source. Third, our study focused on the Nordic populations, which have comprehensive and mainly publicly financed health care systems. In addition, asthma is a heterogeneous disease and the distribution of asthma subtypes is dependent upon the interaction of genetic characteristics and environmental factors. Thereby, our findings may not be generalized to other population.

## Conclusions

Fetal growth and gestational age may play a direct or indirect causal role in the development of childhood asthma.

## Abbreviations

AGA: Appropriate for gestational age; CI: Confidence interval; DMBR: The Danish Medical Birth Registry; ETS: Environmental tobacco smoke; FMBR: The Finnish Medical Birth Register; GA: Gestational age; ICD: The International Classification of Diseases; LGA: Large for gestational age; LMP: Last menstrual period; RR: Relative risk; SGA: Small for gestational age; SMBR: The Swedish Medical Birth Register.

## Competing interests

All authors declare that they have no competing interests.

## Authors’ contributions

XL contributed to data preparation, analysis and interpretation of data, and drafted the manuscript. JO contributed to study design, data analysis, interpretation of the results, and revised the manuscript. EA contributed to study design, data analysis, interpretation of the results, and revised the manuscript. WY contributed to study design, data analysis, the interpretation of the results, and revised the manuscript. SC contributed to study design, data analysis, the interpretation of the results, and revised the manuscript. MG contributed to study design, data analysis, the interpretation of the results, and revised the manuscript. JL contributed to the conception and the design of the study, to data acquisition, data analysis, the interpretation of the results, and revised the manuscript. All authors approved the final manuscript as submitted.
